# Effects of muscle dynamics and proprioceptive feedback on the kinematics and CPG activity of salamander stepping

**DOI:** 10.1186/1471-2202-12-S1-P158

**Published:** 2011-07-18

**Authors:** Jeremie Knuesel, Auke J Ijspeert

**Affiliations:** 1 Biorobotics laboratory, EPFL, Lausanne, 1015, Switzerland

## 

Salamanders typically use a traveling wave of body curvature during swimming, and a standing wave during terrestrial stepping. This pattern is reflected in EMG recordings [1]. A previous model of the salamander central pattern generator (CPG) reproduced these features by means of strong, extensive couplings from limb to axial oscillatory centers, such that limb centers, whenever active, would impose a standing wave of muscle activity in the trunk [2]. However, recent observations have shown that traveling waves of activity in the trunk and limb activity can occur simultaneously, indicating that another mechanism for the generation of the standing wave pattern should be sought. A neuromechanical model of a salamander was used to investigate the possible role of muscle dynamics and sensory feedback in the shaping of the kinematic and CPG pattern. The model uses abstract oscillators for the CPG, a linear Hill model for the muscles, and local proprioceptive feedback. The parameter space was explored systematically in simulations. The walking trot was first simulated without sensory feedback, using a broad range of muscle elastic and damping properties and various muscle activation patterns. Two regions of the parameter space were found to give good locomotion performance: one, with a lower body stiffness, where the kinematics closely followed the muscle activation pattern, and another, with higher body stiffness, where the trunk kinematics approaches a standing wave in spite of a traveling wave of muscle activation (figure 1A). Using muscle parameters from the second region, the effect of local proprioceptive feedback on the CPG was explored with a broad range of ipsilateral and contralateral feedback strengths. A region was found where proprioceptive feedback has a strong modulatory effect on the CPG, leading to an activity pattern resembling a standing wave in the trunk (figure 1B). Proprioceptive feedback also appeared to have a stabilizing effect on the kinematics. The model was then used to simulate aquatic stepping, and could reproduce two important features of this gait: an intersegmental phase lag in between those of swimming and terrestrial stepping, and the presence of passive oscillations in the tail[3].

**Figure 1:**
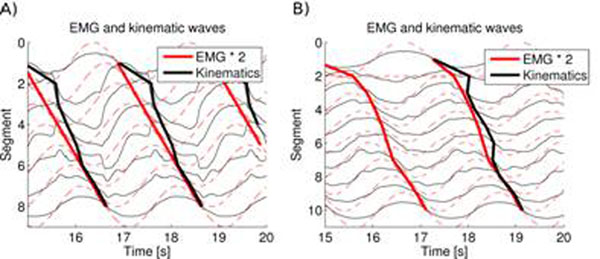
A) kinematics deviating from the EMG pattern in the trunk of an 8-joints model without feedback, illustrating the effect of muscle dynamics. The increased slope of the kinematic line corresponds to motion closer to a standing wave. B) modulation of the CPG by feedback in a 10-joints model (for increased granularity).

## Conclusions

The model suggests that salamanders can modulate their intersegmental coordination by modulating the stiffness of their trunk musculature. Salamanders are known to stiffen their trunk during walking[4]. In the model, such a stiffening leads to a shift in the kinematics away from the traveling wave pattern of swimming, towards the standing wave pattern of the walking trot. With local proprioceptive feedback, this shift can be reflected in the CPG.
